# Late-Onset Systemic Lupus Erythematosus: Is It Really a Benign Disease?

**DOI:** 10.7759/cureus.105262

**Published:** 2026-03-15

**Authors:** Juan Camilo Santacruz, Marta Juliana Mantilla, Sandra Pulido, Carlos Agudelo, Julián Alberto Naranjo, Oscar Vicente Vergara, John Londono

**Affiliations:** 1 Rheumatology, Medicarte IPS, Rionegro, COL; 2 Rheumatology, Centro de Investigación en Reumatología y Especialidades Médicas (CIREEM), Bogotá, COL; 3 Rheumatology, Colsanitas, Bogotá, COL; 4 Rheumatology, Clínica Las Américas, Medellín, COL; 5 Rheumatology, ART Médica IPS, Medellín, COL; 6 Spondyloarthropathies Research Group, Universidad de La Sabana, Chía, COL

**Keywords:** clinical manifestations, elderly patients, immunosenescence, late-onset systemic lupus erythematosus, treatment strategies

## Abstract

Late-onset systemic lupus erythematosus (LO-SLE), typically defined as disease onset at or after 50 years of age, represents a recognized clinical subset of systemic lupus erythematosus with distinctive features. Compared with earlier-onset disease, LO-SLE often presents with a more insidious onset and a somewhat different clinical profile, in which renal and mucocutaneous involvement may be less prominent, whereas serositis, constitutional manifestations, and interstitial lung involvement are more commonly encountered. Immunosenescence is believed to contribute to the pathophysiology of LO-SLE through its effects on both innate and adaptive immune responses. From a serological standpoint, patients in this subgroup may less frequently exhibit anti-double-stranded DNA antibodies and more often demonstrate anti-SSA/Ro and anti-SSB/La antibodies, which can result in clinical and immunological overlap with Sjögren’s disease and create diagnostic complexity. Diagnosis is commonly guided by the 2019 American College of Rheumatology/European Alliance of Associations for Rheumatology classification criteria; however, careful clinical assessment remains essential in older adults due to reduced specificity of antinuclear antibodies and the frequent presence of comorbidities. Management should be individualized, with particular attention to safety, maintaining hydroxychloroquine as the cornerstone of therapy and using glucocorticoids and other immunomodulatory agents cautiously according to disease activity and organ involvement.

## Introduction and background

Late-onset systemic lupus erythematosus (LO-SLE), also referred to as elderly-onset systemic lupus erythematosus (SLE), is defined as disease onset at or after 50 years of age and accounts for approximately 20% of all SLE cases worldwide [1]. Several studies have sought to characterize this subgroup, describing a distinct clinical phenotype in which renal and mucocutaneous manifestations are less frequent, whereas serositis occurs more commonly [2,3].

The pronounced female predominance typically observed in SLE decreases with advancing age. While the female-to-male ratio in early-onset SLE is approximately 9:1, this ratio declines to nearly 7:1 in LO-SLE, with a higher prevalence reported among Caucasian patients [4]. The nonspecific clinical presentation of LO-SLE is frequently associated with diagnostic delays or misdiagnosis, as its manifestations may be incorrectly attributed to physiological changes related to aging [5].

An important consideration in this age group is the impact of multimorbidity on disease outcomes. Aging is associated with an increased prevalence of major comorbidities, including hypertension, diabetes mellitus, and coronary artery disease, which contribute to a higher overall disease burden and increased cardiovascular mortality [6].

Clinically, LO-SLE is more often characterized by cutaneous, neuropsychiatric, hematological, and pulmonary manifestations, including a higher frequency of interstitial lung involvement. In contrast, articular involvement is less prominent compared with that observed in younger patients with SLE [7]. Most patients exhibit positive antinuclear antibodies (ANAs), with a lower frequency of anti-double-stranded DNA antibodies and a notably higher prevalence of anti-Ro/SSA and anti-La/SSB antibodies. These autoantibodies are considerably more common than in younger cohorts and represent valuable diagnostic markers in this population [8].

A distinctive challenge in LO-SLE is the complexity of the differential diagnosis, owing to its overlap with other conditions such as late-onset rheumatoid arthritis, malignancies, tuberculosis, infective endocarditis, polymyalgia rheumatica, giant cell arteritis, and Sjögren’s disease [9].

This narrative review aims to summarize the clinical presentation of LO-SLE, its underlying pathophysiology, autoantibody profile, and current therapeutic approaches, integrating the available clinical evidence to date.

## Review

Pathophysiology

Immunosenescence is a biological process inherent to aging and is characterized by the progressive decline in cellular function, playing a significant role in the pathogenesis of LO-SLE [10]. The core pathophysiology of SLE involves dysregulation of both innate and adaptive immunity, persistent activation of plasmacytoid dendritic cells, increased production of type I interferon alpha, and autoantibody generation by autoreactive B cells [11].

However, recent studies have demonstrated differences in the expression patterns of type I interferon-inducible genes and in the magnitude of the inflammatory response when LO-SLE is compared with early-onset SLE. In older patients, activation of the interferon pathway and neutrophil chemotaxis mechanisms tends to be less pronounced, which correlates with lower acute disease severity and reduced early organ damage, albeit with a higher propensity for chronic disease manifestations and enhanced fibrotic processes [12].

In LO-SLE, immune dysregulation is largely driven by immunosenescence, which is characterized by impaired regulatory T-cell function and a reduced capacity for apoptotic debris clearance. These alterations promote the persistence of autoantigens and sustained immune system activation [13]. Immunosenescence is further associated with a contraction of the T- and B-cell receptor repertoire, accumulation of senescent immune cells, particularly CD4⁺CD57⁺ T lymphocytes, diminished responsiveness to novel antigens, and increased secretion of proinflammatory mediators such as interleukin (IL)-6, IL-8, and tumor necrosis factor-alpha (TNF-α) [14].

Additionally, immunosenescence is accompanied by epigenetic and metabolic alterations, including mitochondrial dysfunction and premature telomere shortening, which contribute substantially to immune dysregulation and chronic low-grade inflammation [15].

Hormonal changes associated with aging, particularly menopause and reduced estrogen production, may partly explain the lower incidence of LO-SLE and the attenuation of female predominance observed in older patients with SLE [16]. Moreover, declining estrogen levels promote lymphopenia through increased reactive oxygen species generation, leading to direct DNA damage and potentially increasing susceptibility to autoimmune diseases in women [17].

**Figure 1:**
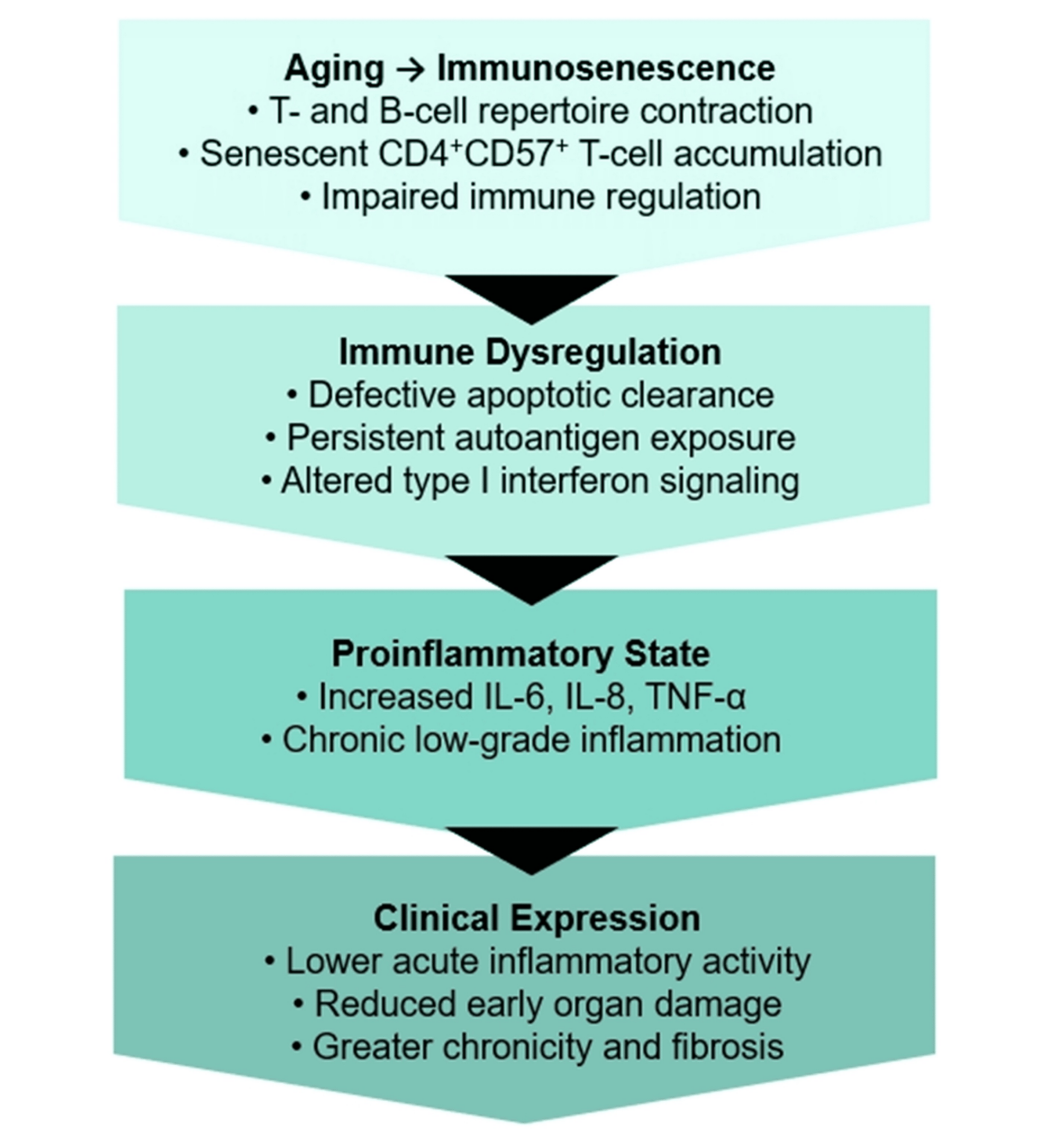
Key pathophysiological mechanisms in late-onset systemic lupus erythematosus. Image credit: Image was created by the authors. IL: interleukin; TNF-α: tumor necrosis factor-alpha

With advancing age, myeloid dendritic cells exhibit impaired antigen-presenting capacity, reduced ability to activate naïve T lymphocytes, and decreased production of IL-12. In addition, these cells show diminished secretion of type I and type III interferons, further contributing to age-related immune dysfunction [18]. Figure 1 summarizes the main pathophysiological aspects of LO-SLE.

Clinical presentation and autoantibody profile

LO-SLE has been proposed to exhibit a distinct clinical behavior, with an initial presentation often dominated by nonspecific constitutional symptoms, which may delay or complicate diagnosis during the early stages of the disease [19]. Commonly reported manifestations include arthralgia that may mimic polymyalgia rheumatica, myalgia, fatigue, serositis, fever, weight loss, cognitive decline, cytopenias, and interstitial lung disease [20].

Although mucocutaneous manifestations are less frequent in elderly patients with SLE, they are not entirely absent and may still form part of the disease spectrum [21]. Photosensitive skin lesions, cutaneous vasculitis, alopecia, Raynaud phenomenon, lymphadenopathy, nephrotic syndrome, and glomerulonephritis occur less frequently in LO-SLE compared with younger-onset SLE [22].

The reported prevalence of interstitial lung involvement in SLE ranges from 3% to 8%, with an increased frequency associated with longer disease duration. Notably, interstitial lung disease appears to be more common in older male patients with LO-SLE [23]. Furthermore, interstitial lung disease does not consistently correlate with classical serological markers of SLE activity, such as anti-double-stranded DNA antibodies or complement consumption. However, a weak association with anti-SSA/Ro antibody positivity has been described, which is clinically relevant when considering possible overlap with Sjögren’s disease, particularly in patients presenting with sicca symptoms or other features suggestive of glandular involvement [24].

A real-world study comparing autoantibody profiles between patients with LO-SLE and those with early-onset SLE demonstrated a lower prevalence of anti-double-stranded DNA antibodies in the LO-SLE group (39% vs. 58%; p = 0.001), as well as reduced anti-RNP positivity (17% vs. 32%; p = 0.02). No significant differences were observed between the two groups regarding anti-Smith, anti-SSA, anti-SSB, lupus anticoagulant, anticardiolipin antibodies, or anti-β2-glycoprotein I antibodies [25]. Additionally, patients with LO-SLE exhibit a higher prevalence of rheumatoid factor positivity and lower rates of hypocomplementemia compared with younger patients.

Hematological involvement is common in LO-SLE, with anemia being the most frequently observed abnormality. Leukopenia and thrombocytopenia may also occur but tend to be less prominent than in early-onset SLE [26,27]. Data regarding neuropsychiatric manifestations in LO-SLE are limited, and their prevalence may be overestimated due to the higher baseline frequency of age-related cognitive and behavioral changes, including cognitive impairment, mood disorders, and acute confusional states. A meta-analysis has reported an overall lower frequency of neuropsychiatric involvement in LO-SLE, with seizures and psychosis being less common, whereas peripheral neuropathy appears to be relatively more frequent [28].

Although the overall prevalence of lupus nephritis is lower in patients with LO-SLE, a multicenter Spanish study reported that approximately 21% of lupus nephritis cases were diagnosed in individuals older than 50 years. This finding may be partly explained by underdiagnosis in older patients, aimed at avoiding renal biopsy-related complications, as well as by a tendency toward less aggressive histological presentations [29]. Reduced activation of the type I interferon pathway and an attenuated renal inflammatory response may contribute to a better response to immunosuppressive therapy, although the risk of chronic progression persists. Lupus nephritis as the initial manifestation of SLE in late-onset disease remains an uncommon and atypical presentation [30].

Table 1 provides a comprehensive comparison of the main clinical differences between early-onset and late-onset SLE, highlighting variations in demographic characteristics, patterns of organ involvement, and disease presentation (Table 1).

**Table 1:** Clinical differences between early-onset and late-onset SLE. SLE: systemic lupus erythematosus

Domain	Early-onset SLE	Late-onset SLE
Age at onset	<50 years	≥50 years
Sex	Female predominance (≈9:1)	Lower female predominance (≈7:1)
Clinical onset	Acute or subacute	Insidious, nonspecific
Constitutional symptoms	Less frequent	More frequent (fatigue, fever, weight loss)
Mucocutaneous involvement	Common (malar rash, photosensitivity, alopecia)	Less frequent
Articular involvement	Common, inflammatory	Less prominent; arthralgias may mimic polymyalgia rheumatica
Serositis	Less frequent	More frequent (pleuritis, pericarditis)
Pulmonary involvement	Rare	Higher prevalence of interstitial lung disease
Renal involvement	Common	Less frequent; when present, similar severity
Neuropsychiatric manifestations	More frequent (seizures, psychosis)	Less frequent; peripheral neuropathy predominates

Diagnosis

LO-SLE does not have specific or distinct classification criteria. Therefore, its diagnosis and classification rely on the same validated criteria used for SLE in general. Among these, the 2019 American College of Rheumatology/European Alliance of Associations for Rheumatology (ACR/EULAR) classification criteria are considered the most appropriate, as they place greater emphasis on immunological findings. This approach is particularly valuable in LO-SLE, where classical clinical manifestations may be less prevalent and atypical presentations are more common [31].

Several studies have shown that patients with LO-SLE accumulate greater organ damage at one and five years after diagnosis compared with younger-onset SLE cohorts. Although the frequency of cutaneous, renal, and central nervous system damage is comparable between groups, LO-SLE is associated with a higher overall disease burden and increased organ damage in specific domains, particularly cardiovascular, ocular, and musculoskeletal systems, as well as a higher prevalence of malignancies [32,33]. It remains unclear whether these findings primarily reflect the effects of aging itself or disease-related factors in elderly patients.

ANA testing retains high sensitivity in LO-SLE; however, its specificity is reduced in older populations, as up to one-third of healthy older adults may exhibit low-titer ANA positivity. Consequently, autoantibody results in this context must be interpreted cautiously and always correlated with the clinical presentation [34].

Exclusion of secondary causes is also essential, particularly drug-induced lupus erythematosus, which is more common in elderly patients and may be reversible. The medications most frequently associated with drug-induced lupus include hydralazine and procainamide [35].

Treatment

Hydroxychloroquine remains the only medication demonstrated to reduce lupus flares due to its pleiotropic effects. These include cardioprotective properties, regulation of glucose metabolism through decreased intracellular insulin degradation and partial inhibition of hepatic gluconeogenesis, as well as antithrombotic effects. Hydroxychloroquine also confers maternal and fetal protection during pregnancy through placental immunomodulatory mechanisms [36]. Additional benefits include prevention of lupus nephritis, reduction in recurrence, and slowing progression to end-stage renal disease, possibly by modulating Toll-like receptor pathways [37,38]. In a large contemporary cohort with systematic retinopathy screening, the cumulative incidence of hydroxychloroquine-associated retinal toxicity was 8.6% after 15 years of exposure, predominantly mild cases, with a clear dose-dependent risk [39].

Glucocorticoids are used for induction therapy during flares, with gradual tapering to the lowest effective dose, ideally ≤5 mg/day of prednisone, to minimize adverse effects such as diabetes, osteoporosis, infections, and myopathy, particularly in older adults [40]. The BLISS trials demonstrated the efficacy of belimumab in SLE and lupus nephritis. Post hoc analyses indicated that patients aged ≥65 years had similar or better responses compared to placebo without increased adverse events, although trials exclusively in this age group are limited due to low incidence [41].

Early introduction of conventional immunosuppressants, including azathioprine, mycophenolate mofetil, and methotrexate, is indicated in moderate-to-severe disease to facilitate glucocorticoid sparing and control disease activity [42]. LO-SLE patients present a lower prevalence of lupus nephritis; however, when nephritis occurs, its severity is comparable to early-onset disease. The overall lower disease activity in LO-SLE is largely due to less frequent renal involvement, rather than a milder renal phenotype [43]. Comparative studies on treatment outcomes in LO-SLE versus early-onset SLE are scarce. One study from Hong Kong found no differences in proteinuria at diagnosis or after 12 months of therapy, suggesting that response to moderate-dose glucocorticoids and immunosuppressants is similar across age groups, though mortality remains higher in LO-SLE [44,45].

Currently, no clinical trials directly compare mycophenolate mofetil versus cyclophosphamide induction therapy in LO-SLE patients with nephritis; therapeutic decisions are typically extrapolated from younger populations, considering comorbidities and drug tolerability. Given its toxicity profile and documented renal response in case reports and small series, mycophenolate may represent a more favorable option in LO-SLE [46]. A single-center study comparing late-onset and classic lupus nephritis observed that LO-SLE patients, predominantly Caucasian, more frequently had overlapping autoimmune disorders such as Sjögren’s disease and antiphospholipid syndrome, a higher prevalence of rheumatoid factor and extractable nuclear antigen antibodies, and a greater incidence of hemolytic anemia and serositis. Time to achieve final renal response was longer in LO-SLE, while relapse frequency was lower [47].

**Figure 2:**
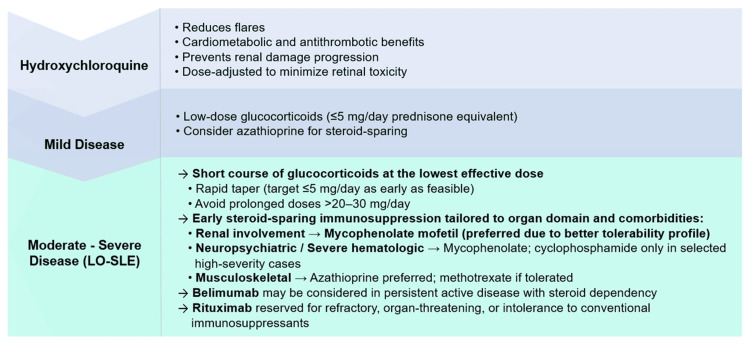
Proposed management strategy for late-onset SLE. Image credit: Image was created by the authors. SLE: systemic lupus erythematosus

A meta-analysis on lupus nephritis confirmed that initial glucocorticoid doses exceeding 40 mg/day were associated with increased infection risk and six-month mortality, reinforcing the need to individualize therapy to balance efficacy and safety [48]. Figure 2 illustrates the proposed treatment approach for patients with LO-SLE.

Prognosis

Although LO-SLE generally exhibits lower disease activity, prevalent comorbidities in this age group contribute to accelerated disease progression, primarily due to greater cumulative organ damage. Age at disease onset itself is an independent risk factor for mortality, reflecting established atherosclerosis, increased vulnerability to cardiovascular events, infections, and osteoporosis associated with late initiation of glucocorticoids [49]. Minimizing glucocorticoid exposure through treat-to-target strategies aimed at achieving remission or a lupus low disease activity state, with prednisone doses ≤5 mg/day or complete withdrawal when feasible, represents a critical prognostic goal [50].

## Conclusions

LO-SLE represents a distinct clinical phenotype, characterized by a more insidious onset and a unique pattern of manifestations. Patients more frequently present with serositis, interstitial lung involvement, and constitutional symptoms, while renal and mucocutaneous involvement are less common. Immunologically, LO-SLE exhibits a characteristic serologic profile, with lower prevalence of anti-double-stranded DNA antibodies and higher prevalence of anti-SSA/Ro and anti-SSB/La antibodies, which often leads to clinical overlap with Sjögren’s disease and complicates differential diagnosis. Despite lower overall disease activity, LO-SLE patients demonstrate greater cumulative organ damage, as measured by the Systemic Lupus International Collaborating Clinics/ACR Damage Index, largely attributable to multimorbidity and prior therapeutic exposure.
